# MUS81 Generates a Subset of MLH1-MLH3–Independent Crossovers in Mammalian Meiosis

**DOI:** 10.1371/journal.pgen.1000186

**Published:** 2008-09-12

**Authors:** J. Kim Holloway, James Booth, Winfried Edelmann, Clare H. McGowan, Paula E. Cohen

**Affiliations:** 1 Department of Biomedical Sciences, Cornell University, Ithaca, New York, United States of America; 2 Department of Biological Statistics and Computational Biology, Cornell University, Ithaca, New York, United States of America; 3 Department of Cell Biology, Albert Einstein College of Medicine, Bronx, New York, United States of America; 4 The Scripps Research Institute, La Jolla, California, United States of America; Stowers Institute for Medical Research, United States of America

## Abstract

Two eukaryotic pathways for processing double-strand breaks (DSBs) as crossovers have been described, one dependent on the MutL homologs Mlh1 and Mlh3, and the other on the structure-specific endonuclease Mus81. Mammalian MUS81 has been implicated in maintenance of genomic stability in somatic cells; however, little is known about its role during meiosis. *Mus81*-deficient mice were originally reported as being viable and fertile, with normal meiotic progression; however, a more detailed examination of meiotic progression in *Mus81*-null animals and WT controls reveals significant meiotic defects in the mutants. These include smaller testis size, a depletion of mature epididymal sperm, significantly upregulated accumulation of MLH1 on chromosomes from pachytene meiocytes in an interference-independent fashion, and a subset of meiotic DSBs that fail to be repaired. Interestingly, chiasmata numbers in spermatocytes from *Mus81^−/−^* animals are normal, suggesting additional integrated mechanisms controlling the two distinct crossover pathways. This study is the first in-depth analysis of meiotic progression in *Mus81*-nullizygous mice, and our results implicate the MUS81 pathway as a regulator of crossover frequency and placement in mammals.

## Author Summary

Failure to undergo faithful meiotic chromosome segregation during mammalian meiosis can result in aneuploidy in the offspring and is a major cause of pregnancy loss and birth defects in humans. One essential component of meiotic prophase I is the exchange of genetic information between maternal and paternal chromosomes, known as recombination or crossing over, and is mediated, at least in part, by the mismatch repair proteins MSH4–MSH5 and MLH1–MLH3. A distinct subset of crossovers in lower organisms is generated by an alternate pathway involving Mus81 endonuclease. Previous studies into the impact of *Mus81* mutations in mice revealed no adverse effect on the fertility of these animals. In this study, we report subtle, yet significant, defects in meiotic progression in male and female *Mus81* mice, coupled with intriguing results showing that MUS81 protein is essential for crossover control in mammals. MUS81 appears to be required for correct localization of MLH1–MLH3 complexes to paired homologous chromosomes, however, not for the maintenance of physical crossovers, visualized as chiasmata. These results show a complex interplay between the MUS81 and MLH1–MLH3 pathways for generation of crossovers and, as such, are critical to the further understanding of the intricacies of crossover control with a view to reducing meiotic error rate in humans.

## Introduction

Meiosis is a tightly regulated and essential process that results in the generation of gametes containing the correct haploid chromosome complement. The defining events of meiosis occur during prophase I, including the pairing of and physical association between, homologous chromosomes (synapsis), accompanied by exchange of genetic information (recombination) between these chromosome pairs. These meiotic regulatory processes are highly conserved from yeast through to humans. Recombination is initiated by the formation of double-strand breaks (DSBs), an event that is catalyzed in most eukaryotic species by the meiosis-specific endonuclease Spo11 [1], and then processed via the DSB repair pathway [2]. The process of DSB repair in mammals appears to utilize pathways similar to that seen in lower eukaryotes, such as *S. cerevisiae* [3],[4], and results in either crossover (CO), which involves exchange of flanking DNA markers between the homologs, or non-crossover (NCO), in which the flanking DNA remains unchanged [3]. COs are physically manifested as chiasmata, which tether homologous chromosomes together to ensure correct segregation at the first meiotic division.

In many organisms, the majority of COs are subject to regulation by a phenomenon known as interference, a process by which the presence of a CO on a chromosome greatly decreases the chances of a second CO occurring nearby on the same chromosome [5]. Thus, COs susceptible to interference remain widely spaced, and are less likely to cause problems during segregation. In mammals, interference is generally measured between MLH1 foci at pachynema, so that the interference measurement in this regard is between MLH1 events, rather than between COs [6],[7].

In *S. cerevisiae*, at least two CO pathways have been described, one dependent on the MutL homologs, Mlh1 and Mlh3, and the other on the structure specific endonuclease, Mus81 [8]. The majority of COs are processed by the interference-dependent Msh4–Msh5 and Mlh1–Mlh3 pathway (Class I CO)[8], while a second class of interference-independent CO (Class II CO) [8],[9] are thought to be processed by the alternative Mus81-Mms4 pathway . There is some evidence for a third pathway which, in the absence of Msh4–Msh5 and Mms4, generates COs in *S. cerevisiae* [10],[11]. In *S. pombe*, Mus81-Eme1 generates most, if not all, COs [12] and these CO events are not subject to interference [13].

The exact mechanism of Mus81 action remains unclear, and has been the subject of some controversy. Mus81 may act early in the DSB repair pathway, following the point of strand invasion, or later, during Holliday Junction (HJ) resolution [14],[15],[16],[17]. More recent evidence suggests Mus81 is able to cleave intact single HJs in *S. pombe* [15],[17], the intermediates of recombination in that species, although these structures are thought less likely to be the substrates for Mus81 in budding yeast and higher eukaryotes [15]. However, *Drosophila melanogaster* MEI-9 protein, an XPF-type endonuclease similar to MUS81, has been implicated in Holliday junction resolution and DSB repair in fruit flies [18].

In *Arabidopsis thaliana*, both the Class I (interference-dependent, AtMlh1–AtMlh3 regulated) and Class II (interference-independent, AtMus81-regulated) CO pathways have been described [19]. In *Atmus81* mutants, the number of physical COs is maintained at wild-type levels, even in the absence of this proposed alternate AtMus81-dependent CO pathway [20]. However, in the *Atmsh4/mus81* double knockout, COs are reduced compared to the *Atmsh4* single mutant [20], indicating that AtMus81 plays a minor role in generating a subset of meiotic crossovers in wild-type plants. Residual chiasmata are seen, even in the *Atmsh4/mus81* double mutant, suggesting that a third pathway to generate meiotic COs is also present in higher eukaryotes.

In mice, the existence of an alternative, MLH1–MLH3–independent, pathway has yet to be demonstrated. In the absence of *Mlh1* or *Mlh3*, chiasmata numbers are reduced approximately 10 to100-fold, but are not removed entirely [6],[21],[22]. In both male and female *Mlh1^−/−^* mice, diakinesis chromosome preparations reveal mostly univalent homologs [6],[21], with the level of chiasmata in *Mlh1* oocytes severely reduced compared to WT (1.9 and 24.1 average chiasmata per cell respectively) [21]. Diakinesis stage spermatocytes from *Mlh3^−/−^* mice show a similar depletion in chiasmata [22]. In both *Mlh1* and *Mlh3* knockout mice, a subset of 5–10% of wild-type (WT) CO persist at the *Psmb9* recombination hotspot [23],[24], indicating that the MLH1–MLH3 pathway is responsible for the majority of CO events, but also suggesting that alternate CO pathways exist in mammals.

Previous analysis of two different strains of *Mus81* disrupted mice revealed increased DNA damage and susceptibility to DNA cross-linking agents such as mitomycin-C, Curiously, meiotic progression in these mutants appeared to be normal [25],[26]. Here we provide the first detailed analysis of meiotic progression in *Mus81* null mice, and reveal that mutant males show reduced sperm number, consistent with spermatogenic cell arrest during meiosis. While some germ cells can overcome this meiotic disruption, others cannot and do not progress through meiosis. These results demonstrate the disruption of normal meiotic progression in *Mus81* mice, which leads to proposing the existence of a new crossover pathway in mammals, which has wide reaching implications for mechanisms of crossover control and a direct role for MUS81 in meiotic DSB repair.

## Results

### *Mus81* Homozygous Mutant Males Show Reduced Testis Size and Sperm Numbers

**Figure 1:**
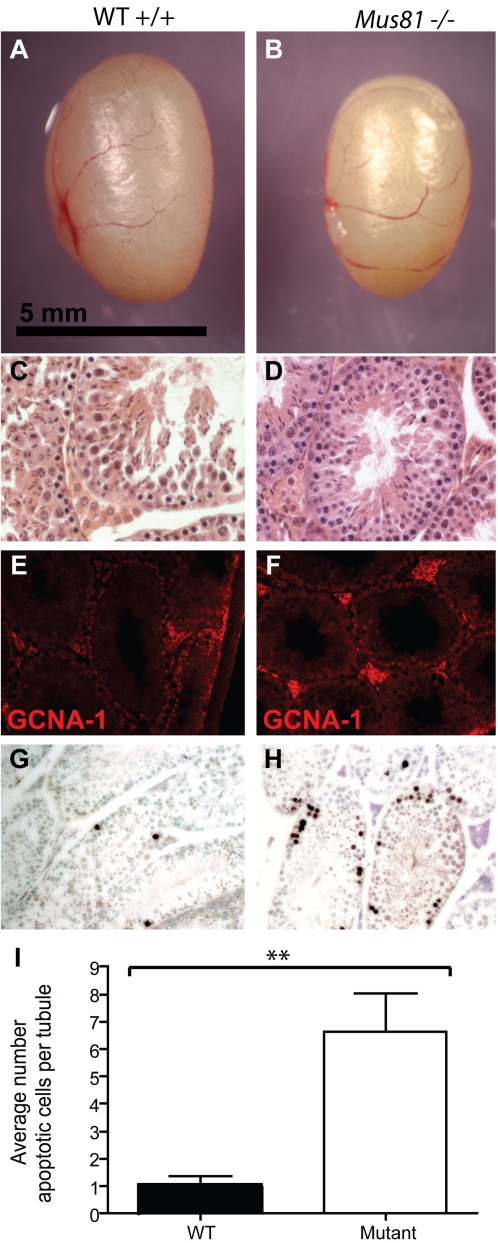
*Mus81^−/−^* male mice have reduced testes size and sperm number. Testes from WT (A) and *Mus81^−/−^* (B) mice were removed, weighed and photographed. Scale bar 5 mm. C, D) Respectively, WT and *Mus81^−/−^* testes sections stained with H&E. E, F) WT and *Mus81^−/−^* testes sections stained with antibody to GCNA-1 raised in rat (red). G, H) WT and *Mus81^−/−^* testes sections TUNEL stained for apoptotic cells (dark brown) and counter stained with methyl green. I) Average number of apoptotic cells per tubule in WT and *Mus81^−/−^* testis sections, which are significantly different between the two genotypes (P = 0.0073).

*Mus81* null mice show defects in meiotic progression, manifested by reduction in testis size (Figure 1A, B) and a decrease in mature spermatozoa within the epididymides (Table 1). In line with previous reports, this reduced reproductive function is not sufficient to render the mice infertile [25],[26]. Differences in gross testis organization between *Mus81^+/+^* and *Mus81^−/−^* males are not obvious from H&E staining (Figure 1C, D) [26]. Early meiotic progression appears unaffected in *Mus81^−/−^* males, with germ cell nuclear antigen-1 (GCNA-1) staining of spermatogonia and early spermatocytes being similar in mutant and WT males (Figure 1E, F). However, the cell density of the seminiferous epithelium appears reduced in the mutant testes compared to that of WT litter mates, accompanied by a significant increase in the number of apoptotic cells (P = 0.0073) (Figure 1G–I). The location of apoptotic cells in the testes of *Mus81^−/−^* males, from pachynema to metaphase, indicates a loss of spermatocytes during prophase I, however a proportion of cells escape this apoptosis since many meiosis II spermatocytes and post-meiotic spermatids are observed (Figure 1C, D).

**Table 1:** *Mus81* mice show reduced testis size and sperm number.

Mouse Genotype	Average single testis weight (mg)	% of WT	% of *Mus81^−/−^*	Average sperm number (per ml)	% of WT
*Mus81^+/+^*	102±16.7 mg	-	-	2.3×10^7^	-
*Mus81^−/−^*	68.5±1.2 mg	65	-	0.9×10^7^	40
*Mus81^+/+^.Mlh3^−/−^*	44.4±3.9 mg	45	65	0	0
*Mus81^−/−^.Mlh3^−/−^*	28.3±0.7 mg	27	41	0	0

Mice of different genotypes show varying testis weights and sperm numbers.

### Analysis of Prophase I in *Mus81* Mutant Males Reveals Persistence of Unrepaired Double Strand Breaks and Persistence of the Early Meiotic Nodule Component, BLM

**Figure 2:**
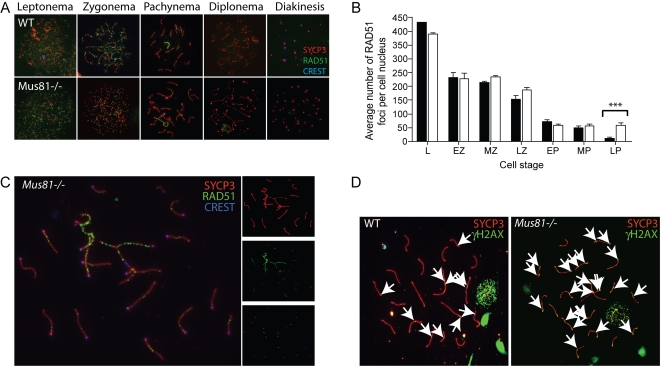
Meiotic recombination analysis in WT and *Mus81^−/−^* spermatocytes by immunofluorescent staining and diakinesis spreads. A, B) RAD51 staining persists in late pachynema. A) WT (top panel) and *Mus81^−/−^* (bottom panel) spermatocytes stained with antibodies against SYCP3 (red), RAD51 (green) and CREST autoimmune serum (blue) in the five substages of Prophase I; Leptonema, Zygonema, Pachynema, Diplonema and Diakinesis. B) RAD51 focus numbers are not statistically different in *Mus81^−/−^* spermatocytes (shown as white bars) compared to WT (black) except in late pachynema, where they persist in *Mus81^−/−^* (significant difference shown by the asterisk). C) *Mus81^−/−^* spermatocytes stained with antibodies against SYCP3 (red), RAD51 (green) and CREST autoimmune serum (blue) during late pachynema. D) WT (left panel) and (*Mus81^−/−^*) spermatocytes were stained with antibodies to SYCP3 (red) and phosphorylated γH2AX (green). γH2AX foci are highlighted by the arrows.

To study the progression of synapsis and recombination events during prophase I, chromosome spreads were prepared as described previously [27]. Indirect immunofluorescence on chromosome spreads was performed to localize meiotic proteins SYCP3, phosphorylated H2AX (γH2AX), and RAD51 on chromosome spread preparations of spermatocyte nuclei (see materials and methods). SYCP3 is a protein that localizes to the lateral elements of the symaptonemal complex during meiosis, and allows the visualization of chromosome cores. Histone H2AX is a ubiquitous member of the histone H2A family that, upon DSB induction, is rapidly phosphorylated on serine 139. This phosphorylated form of H2AX, referred to as γ-H2AX, also localizes to regions of silenced chromatin and is thus used in meiotic cells to mark regions of DSBs, asynapsis and the sex body [28]. Processing of DSBs in early prophase I requires the participation of RecA homologs, RAD51 and DMC1 [29], components of early meiotic nodules in mice [30],[31],[32]. RAD51, forms a nucleofilamentous structure along single stranded DNA and facilitates strand invasion in the early stages of DSB repair during leptonema and zygonema, and once synapsis occurs, disappears from the chromosome cores, indicating progression of repair events beyond strand invasion [33]. In *Mus81^−/−^* males, RAD51 accumulates normally on meiotic chromosomes, but the foci persist to late pachynema in mutant animals, indicating that not all meiotic DSBs are repaired correctly (Figure 2A, B). In addition to these small regions of localized RAD51 staining, some late pachytene spermatocytes (<5% total) show larger regions of asynapsis, indicating a role for MUS81 in correct pairing of homologous chromosomes in a subset of spermatocytes, as visualized by the persistence of both RAD51 and γ-H2AX stains on autosomes in late pachynema (Figure 2A–D).

**Figure 3:**
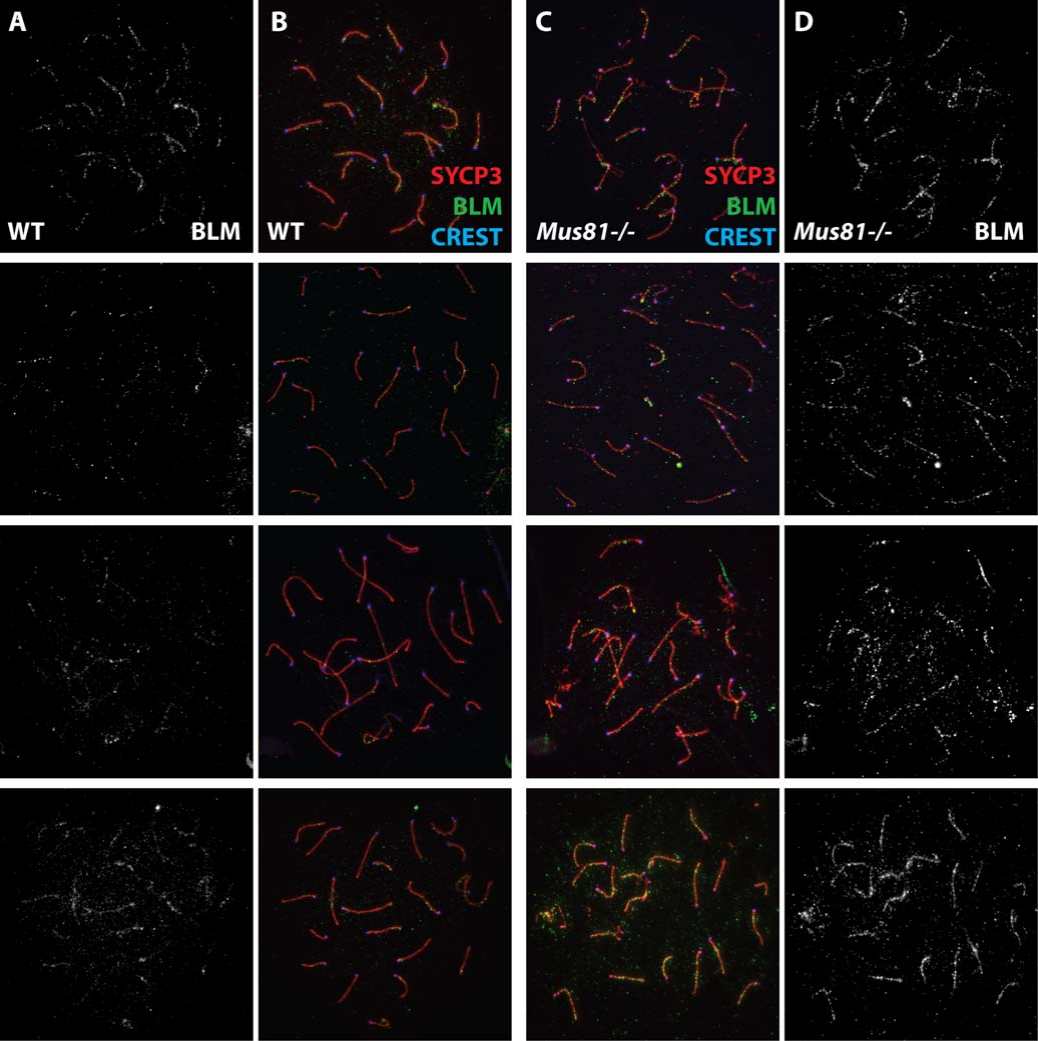
BLM localization persists into late pachynema in *Mus81^−/−^* spermatocytes. WT (column A, B) and *Mus81^−/−^* (column C, D) pachytene spermatocytes were stained with antibodies raised against SYCP3 (red), BLM (green) and CREST autoimmune serum (blue). BLM focus number is higher in late pachytene cells in the mutant as opposed to WT cells, as can be seen when visualizing the BLM only image (white) (columns A, D).

Late pachytene spermatocytes also show increased accumulation of the RecQ helicase Bloom syndrome mutated (BLM), the mammalian ortholog of yeast Sgs1. Previous studies have demonstrated that BLM accumulates on chromosome cores during Prophase I in WT spermatocytes, appearing early in zygonema and gradually declining through mid-pachynema [34],[35]. In the current study, a similar increase in BLM foci is observed at zygonema in WT spermatocytes, before decreasing to a few foci in mid-late pachynema (Figure 3A, B). In spermatocytes from *Mus81^−/−^* males however, BLM focus numbers rise during zygonema but then remain elevated above WT levels throughout the entire pachytene stage, with increased foci being distributed along all chromosomes in an individual nucleus (Figure 3C, D).

### Absence of MUS81 in Spermatocytes Results in Increased Accumulation of MLH1–MLH3 on Pachytene Chromosomes and Reduced Interference

**Figure 4:**
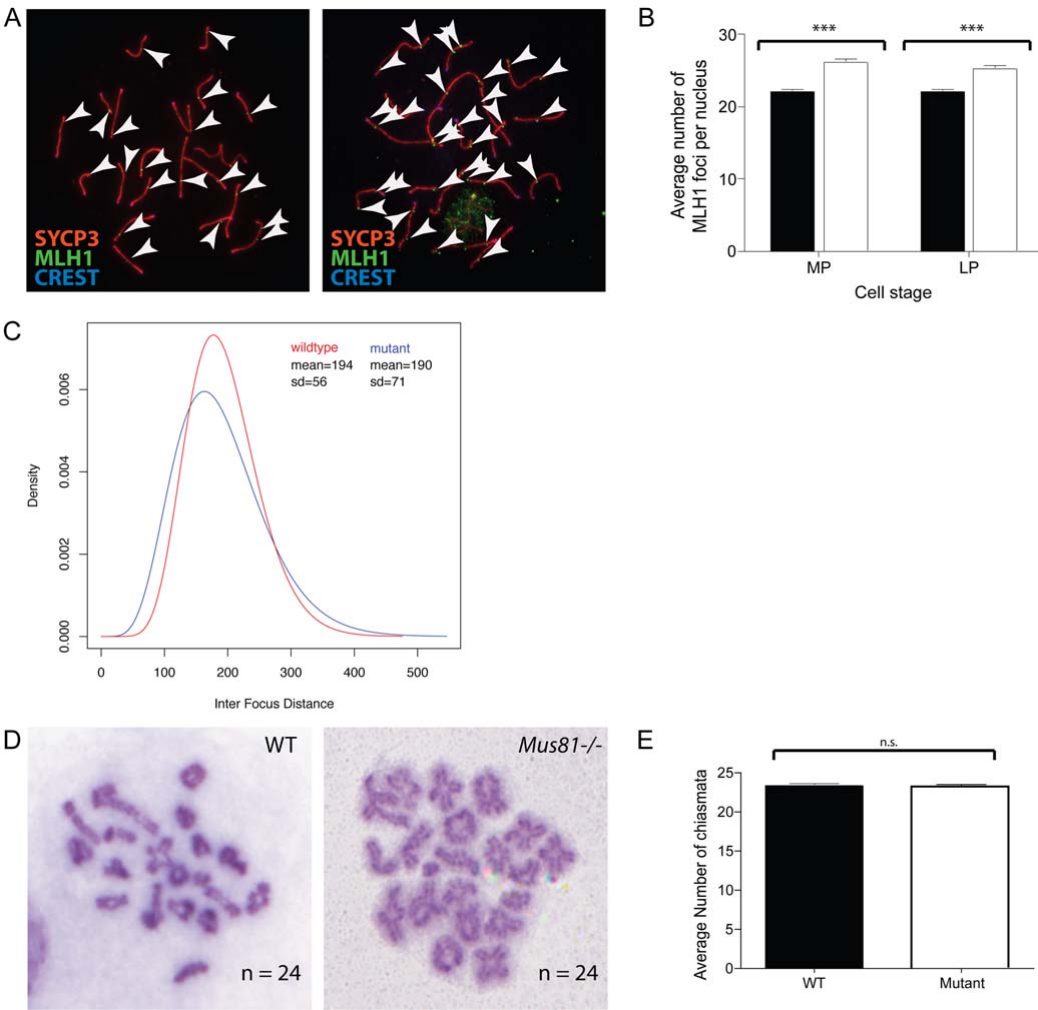
MLH1 accumulation is upregulated in *Mus81* nulls, while interference is reduced. A, B) MLH1 focus numbers are increased in *Mus81^−/−^* spermatocytes compared with WT. A) Chromosome spreads from WT (left panel) and *Mus81^−/−^* (right panel) spermatocyte stained with antibodies against SYCP3 (red), MLH1 (green) and CREST autoimmune serum (blue). White arrows show the positions of MLH1 foci for clarity. B) Average MLH1 focus numbers counted in mid pachynema (MP) and late pachynema (LP). Statistically significant increases in *Mus81^−/−^* counts (white) compared with WT (black) are shown by the asterisks. C) Interference is reduced in the mutant (blue) compared to the WT (red). D, E) Chiasmata counts on cells at diakinesis of prophase I for WT (left) and *Mus81^−/−^* (right) mice, chiasmata numbers for each cell are shown, as well as average counts for WT and *Mus81^−/−^*.

The accumulation of MLH1 and MLH3 on meiotic chromosome cores in early to mid pachynema occurs at a time when, it is thought, the final number of crossover events is set. Spermatocytes from *Mus81^−/−^* males show a significant increase in the number of MLH1 foci, from 22.07±0.39 (mean±s.d.) in spermatocytes from wild-type males to 25.26±0.52 in spermatocytes from *Mus81^−/−^* males, reflecting an average increase of 3.1 foci per cell during late pachynema (P<0.05: Figure 4A, B). Importantly, the increase in MLH1 focus numbers is not associated with changed in the length of the synaptonemal complex. In contrast to the normal cohort of MLH1 foci observed in mouse spermatocytes [36], these additional foci appear to be interference-independent. When inter-focus data were fitted to a gamma distribution [37], the level of interference between MLH1 foci in spermatocytes from *Mus81^−/−^* (12.7±1.8 s.d.) was reduced with respect to that of WT males (15.9±2.9 s.d.) (P = 0.1). However, since this measurement only takes in to account those chromosomes exhibiting more than one MLH1 focus (long chromosomes), an alternative censoring technique was employed to estimate interference on chromosomes with only one focus (hence, shorter chromsomes). Censoring is a statistical method used when the value of an observation is only partially known. More specifically, right-censored data refers to that in which a data point is above a certain level, the extent of which is unknown (see Materials and Methods). When using the right-censored data, the fitted gamma distributions are significantly different when compared using a likelihood ratio test (P<0.0001; Figure 4C), indicating that interference is reduced in the mutant.

In contrast to the increased numbers of MLH1 (Figure 4A–C) and MLH3 (data not shown) focus numbers at pachynema, MSH4 focus numbers were not significantly different between WT and *Mus81^−/−^* cells in mid-late zygonema (P = 0.34), indicating no change in the recruitment of the MSH4–MSH5 heterodimer to meiotic nodules in early prophase I (data not shown).

### Normal Chiasma Counts in Spermatocytes from *Mus81^−/−^* Males

In view of the increased MLH1–MLH3 frequency in *Mus81^−/−^* spermatocytes, and given the role of these proteins in marking ultimate sites of the majority of CO in other higher eukaryotes [38],[39], we asked whether the increased MLH1 foci in *Mus81^−/−^* spermatocytes leads to an increase in physical crossovers. To this end, spermatocytes were prepared for diakinesis analyis by incubation in hypotonic buffer followed by methanol-acetic acid fixation and spreading (see materials and methods). Surprisingly, there was no significant difference in the number of chiasmata in WT and *Mus81^−/−^* air-dried diakinesis spreads (P = 0.64: Figure 4D, E) indicating normal rates of crossing over in the absence of MUS81.

### Normal Ovarian Development in *Mus81^−/−^* Females Despite Elevated MLH1 Counts in Prophase I Oocytes

**Figure 5:**
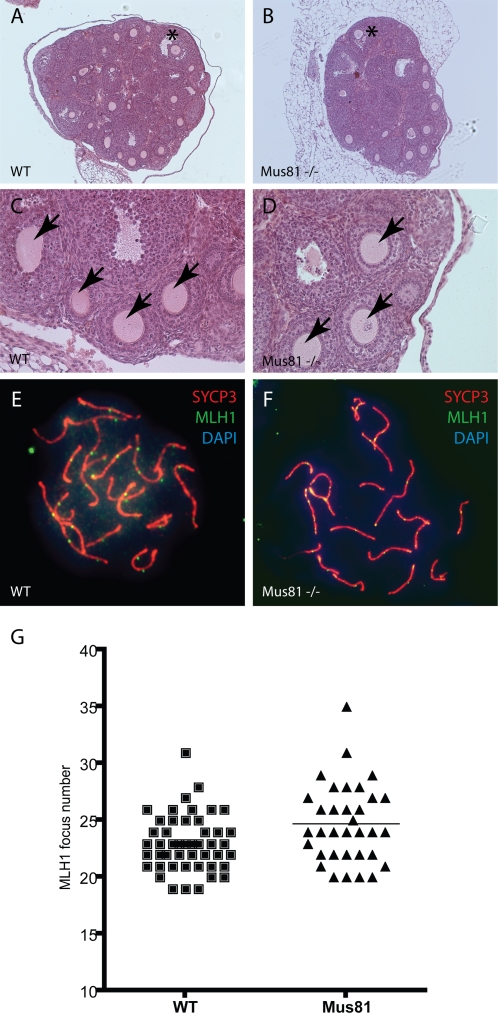
Meiotic Analysis in *Mus81^−/−^* females. WT (A, C) and *Mus81^−/−^* (B, D) day 22 pp ovaries sectioned and stained with H&E show normal oocyte development. Secondary (arrows) and antral (asterisk) follicles are present in both mutant and WT ovaries. MLH1 staining of WT (E) and *Mus81^−/−^* (F) oocytes from day e18.5 embryos show increased MLH1 foci in *Mus81^−/−^* (G).

*Mus81^−/−^* females have been previously reported to show WT levels of fertility [26]. Ovaries from adult *Mus81^−/−^* females appear histologically similar to wild-type females of the same age (Figure 5A, B). Closer examination of the ovaries of wild-type (Figure 5C) and *Mus81^−/−^* (Figure 5D) females reveals oocytes at all stages of follicular development, including primary and secondary follicles (arrows in Figure 5D), as well as those in the antral stages (asterisk in Figure 5A,B). The ability of *Mus81^−/−^* females to produce normal litter sizes (data not shown and McPherson et al. 2004) indicates that these follicles are viable and can give rise to healthy gametes. However, like their male counterparts, MLH1 counts at pachynema in oocytes from *Mus81^−/−^* females are significantly higher than in WT oocytes (n = 34 and 48 respectively, mean foci per cell 24.74±3.47 s.d. and 23.00±2.47 s.d., respectively P = 0.0097, Welch's correction test performed to compare variances: Figure 5E–G), which is an average increase of 1.74, slightly fewer than those seen in *Mus81^−/−^* spermatocytes, but nonetheless significant.

### Analysis of *Mus81: Mlh3* Double Null Reveals a Reduction in Chiasmata between *Mlh3* Single and *Mus81: Mlh3* Double Mutants

**Figure 6:**
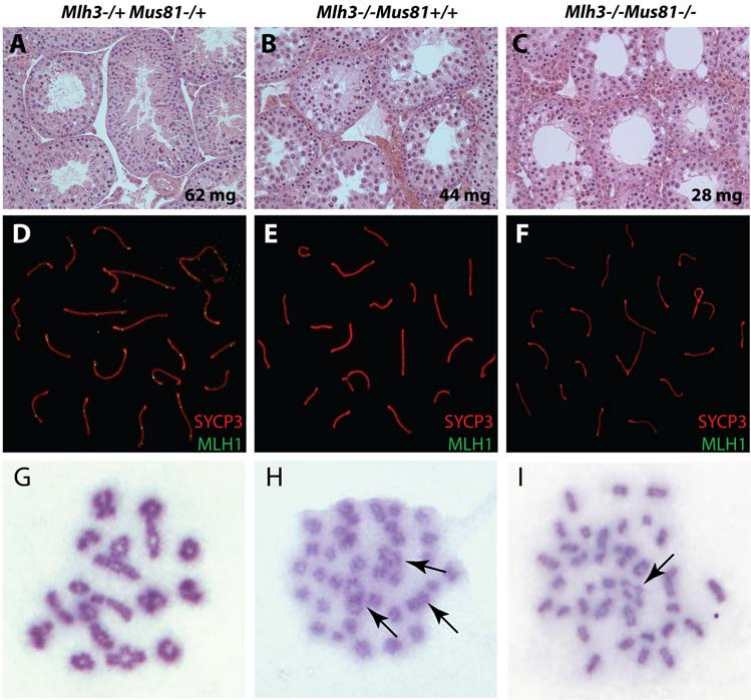
Analyses of *Mus81^−/−^/Mlh3^−/−^* double null mice. *Mlh3^−/+^:Mus81^−/+^*(A), *Mlh3^−/−^:Mus81^+/+^* (B) and *Mlh3^−/−^:Mus81^−/−^* (C) testes sections were analyzed by H&E staining. Chromosome spreads from the same mice stained with antibodies against SYCP3 (green) and MLH1 (red) (D–F). Diakinesis spreads show normal levels of chiasmata in the *Mlh3^−/+^:Mus81^−/+^* mice, reduced levels in the *Mlh3^−/−^:Mus81^+/+^* mice and only a residual level of chiasmata in the *Mlh3^−/−^:Mus81^−/−^* double knock-out mice (G–I). Arrows show the positions of the residual crossovers.

To ascertain if MUS81 acts in a separate crossover pathway to MLH3, both single and double mutants in the two genes were analyzed for meiotic progression and chiasmata frequency. Testis sizes of the *Mus81^−/−^* and *Mlh3^−/−^:Mus81^−/−^* mice were reduced compared to wild-type males (Table 1), and this is evident by the reduced cellularity of the seminiferous epithelium in the double mutant males (Figure 6D–F). Both the single *Mlh3^−/−^* and double *Mus81^−/−^:Mlh3^−/−^* mutants had no mature spermatozoa within their epididymides (Table 1), and no overt differences were observed in localization patterns for different prophase I markers between the two genotypes (MLH1, Figure 6G–I). Chiasmata were reduced in the *Mus81^−/−^:Mlh3^−/−^* double mutant males, compared to the *Mlh3^−/−^* single mutant males and both were severely reduced when compared to WT (average chiasmata counts per cell were WT 23.79 ±1.78, *Mlh3^−/−^* 1.79 ±0.80, *Mus81^−/−^: Mlh3^−/−^* 0.48 ±0.70: Figure 6J–L, with the counts from *Mlh3^−/−^* single null and *Mus81^−/−^: Mlh3^−/−^* being significantly different p<0.0001). Thus, it appears that MUS81 and MLH3 generate COs by independent pathways, as exemplified by the residual crossovers in the *Mlh3* null mice, and that there is a possible third pathway for generation of COs, due to the presence of a small number of remaining COs in the double nulls.

## Discussion

These studies demonstrate that deletion of *Mus81* in mice has severe consequences for meiotic progression. Male *Mus81^−/−^* mice exhibit reduced testis size and epididymal sperm numbers, coupled with increased seminiferous tubule apoptosis that is not confined to a single stage of spermatogenesis. Meiotic DSB repair appears normal in the early stages of prophase I, as assessed by the accumulation of RAD51 and MSH4 on meiotic chromosomes, but by pachynema there is a significant increase in the numbers of MLH1 and MLH3 foci on the chromosome core of both spermatocytes and oocytes from *Mus81* nullizygous animals. Since recombination rate (and hence MLH1 focus frequency) is tightly associated with synaptonemal complex length [40], it is important to note that these increases in MLH1–MLH3 foci are not associated with changes in synaptonemal complex length. Moreover, we see reduced interference amongst MLH1 foci in spermatocytes from *Mus81^−/−^* males, indicating that the additional MLH1–MLH3 events are not subject to the normal strict regulation of crossover placement that is essential to ensure appropriate segregation of chromosomes at the first meiotic division. Late in pachynema, spermatocytes from *Mus81^−/−^* males show persistent and upregulated localization of BLM helicase indicating a failure to repair DSBs appropriately and/or the presence of aberrant DNA structures in late prophase I. Interestingly, however, the persistence of BLM is associated with normal chiasmata numbers at diakinesis in *Mus81^−/−^* males, despite the increase in MLH1–MLH3 foci observed at pachynema. The data herein represent the first comprehensive analysis of the effects of *Mus81* mutation on meiotic progression and DSB repair in mice and demonstrate the possible existence of a second CO pathway in mammalian meiosis. Moreover, these data indicate important cross-regulatory mechanisms between the two CO pathways in mammals.

In *S. cerevisiae* and *S. pombe*, the synthetic lethality of *mus81.sgs1* (or *mus81.rqh1* for *pombe*) double mutants [12],[41],[42],[43] can be rescued by deletion of *rad51* [44],[45] , suggesting that the removal of homologous recombination events can prevent the accumulation of toxic recombination events during vegetative growth. Extensive studies in *S. cerevisiae* have also demonstrated the requirement in lagging-strand replication for additional interactions between Mus81 and Sgs1 that are independent of homologous recombination [44], and the role of Mus81 in this scenario is, in fact, primarily independent of Rad51 [46]. Li and Brill [46] have proposed a model in which Mus81 cleaves 3′-flaps present on the lagging strand to result in polymerase-directed repair in most cases. Alternatively, the 3′ end may become recombinogenic, resulting in Rad51-directed double Holliday junction (HJ) formation and subsequent activation of the major repair pathway involving Sgs1. Additionally, a minor pathway may be initiated by Rad51-mediated strand displacement and subsequent Mus81 activity [46]. Thus, in this model, Mus81 functions to remove 3′ flaps prior to Rad51-induced double HJ formation.

Similar models have been proposed during meiosis in *S. cerevisiae*, supported by the observations that double HJs do not accumulate in *mms4* mutant strains [8],[47], that the reduction in crossing over in this strain is extremely modest (<1.8-fold), and that expression of a bacterial HJ resolvase fails to suppress the *mus81* meiotic phenotype [8].

Despite the lack of evidence supporting a role for Mus81 in double HJ resolution during meiosis in yeast, mutants for *mus81* or *mms4* show delayed repair of DSBs and appearance/disappearance of recombination intermediates. This involvement in later stages of DSB repair may be explained by the model proposed by De Los Santos *et al*, in which Mus81/Mms4 are required for a subset of recombination intermediates in which over-replication of the displaced invading strand following D-loop formation (for details see [8]). Taken together, these models all suggest a mechanism by which Mus81 functions upstream (and independently of) Rad51, presumably through cleavage of 3′ flaps, to effect DNA repair but, in addition, can act downstream of Rad51 to mediate repair involving Holliday junction intermediates (but again through its action on 3′ flaps).

Our data on MUS81 function in mice is congruent with both possibilities presented above. An early role for MUS81 in prophase I in mice is indicated by the loss of meiotic cells from leptonema onwards, and by the observation of increased RAD51 staining associated with regions of asynapsis and synaptic disruption in zygonema (Figure 2C), although it must be noted that homolog association and synapsis are unaffected in *S. cerevisiae mms4* mutants [8]. However, the fact that RAD51 focus numbers are largely unaffected in *Mus81* nullizygous mice, together with the observation that MSH4 focus frequency is normal in these animals, would argue that the early stages of DSB repair are unaffected by the absence of MUS81. It is possible that the subset of DSB events that are destined to become substrates for RAD51 is unaffected by the loss of MUS81 and that only those MUS81-dependent DSBs are then left unrepaired, perhaps becoming the substrate for DNA mismatch repair processes. However, this is inconsistent with our observation that the increase in MLH1/MLH3 focus numbers does not occur until mid-to-late pachynema, much later than the appearance of these aberrant DNA structures. Moreover, that the additional foci of MLH1 are equally represented by additional foci of MLH3 implies that these extraneous MutL heterodimers are of the MLH1/MLH3 variety (involved in recombination events) and not of the MLH1/PMS2 variety (involved in canonical mismatch repair), although analysis of PMS2 localization is prevented by the absence of a functional antibody. Nonetheless, we cannot exclude the possible involvement of a non-canonical mismatch repair complex in these events, nor can we dismiss the possible importance of other repair pathways in these processes. Alternatively, such aberrant structures, which fail to be processed by MUS81 in early leptonema, could proceed through prophase I to pachynema, whereupon they become substrates for MLH1/MLH3 accumulation (giving rise to the increased focus numbers for these MutL homologs). The lack of an appreciable increase in MSH4 foci would argue against this possibility, although the predicted increased may be too small to be evident above the normal level of MSH4 accumulation.

Previous studies from other organisms have shown Mus81 to be important in the processing of interference-independent COs [8],[9] We believe non-interfering COs might be generated in the same way in mice, as *Mus81^−/−^* males show irregularities in processing of late recombination intermediates, characterized by a significant increase in interference-independent MLH1 foci. Intriguingly, this increase in MLH1 does not correspond to an increase in chiasmata in the *Mus81^−/−^* mice, when compared with WT (discussed below). Thus, *Mus81* deletion represents the first single null mutant in which an increase in MLH1 foci is *not* correlated with increased SC length and, more importantly, in which the increase in MLH1 focus numbers does *not* result in an increase in the final tally of chiasmata. That these increased MLH1 foci are associated with similar increases in MLH3 suggests that the MLH1 function at these sites is one of recombination rather than of DNA mismatch repair (which would utilize PMS2). Unfortunately, the lack of a MUS81 antibody that detects the protein on chromosome spreads precludes detailed analysis of MUS81 localization in spermatocytes (Holloway and Cohen, unpublished data). These observations point to alterations in crossover control at the level of at least two distinct, but converging, recombination repair pathways and not to regulation of crossover frequency by the SC *per se*.

**Figure 7:**
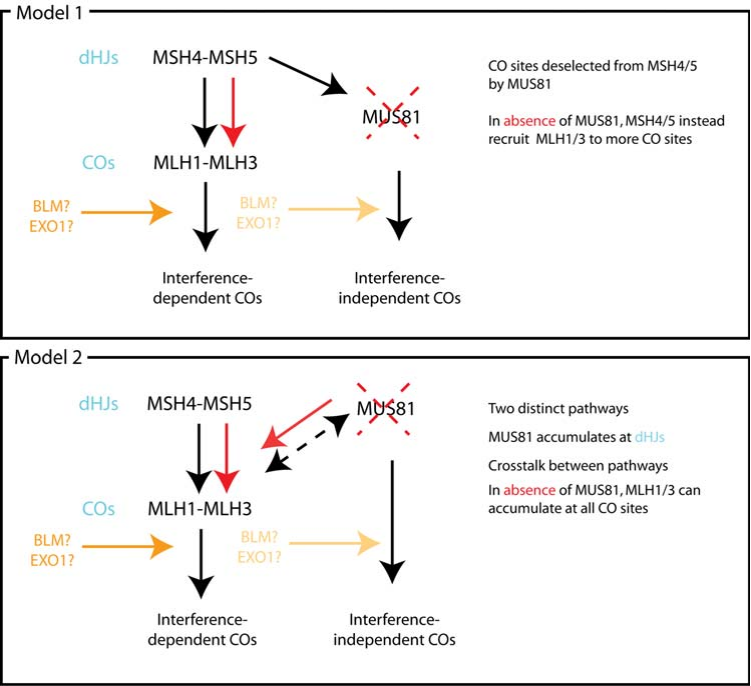
Two possible models for late action of MUS81 in mammalian meiosis.

Given the late prophase I increase in MLH1–MLH3 foci in *Mus81* nullizygous animals, it is possible that MUS81 may play both an early and a late role in recombination events in the mouse, the two possibly being delineated by RAD51-independence versus RAD51-dependency as suggested by Li and Brill [46]. For the later, RAD51-dependent function, there are two possible models for the interplay between MSH4–MSH5, MUS81 and MLH1–MLH3 in generating COs (Figure 7). The first posits that MSH4–MSH5 will bind model dHJs [48], recruit MLH1–MLH3 to the majority of CO sites, while recruiting MUS81 to the remaining subset of CO sites (or the presence of MUS81 at this subset prevents MLH1–MLH3 recruitment here). In the absence of MUS81, MSH4–MSH5 directs all COs to be processed by MLH1–MLH3. There is evidence from tomato that MLH1-positive COs and MLH1-negative COs may arise from the same early precursors [39]. Moreover, data from mice and humans show that genetic maps are consistently longer than those estimated from MLH1 focus counts, suggesting that MLH1 foci do not represent all the physical COs generated during meiosis [7],[49],[50]. This model, however, would require physical interaction between MSH4–MSH5 and MUS81, a prerequisite for which there is no published data. In addition, evidence from *A. thaliana* suggests that, if this were the case, *Atmsh4* mutants and *Atmsh4.mus81* double mutants would show the same number of residual chiasmata, which is not the case [20].

The second model assumes that MSH4–MSH5 and MUS81 act in separate pathways, between which there is some degree of crosstalk. This model also assumes that mammalian MUS81 acts at HJs *in vivo*, which has been shown *in vitro* using recombinant human MUS81 [16]. The presence of MUS81 may then prevent MLH1–MLH3 binding to a subset of CO sites, resulting in processing of these COs down a MUS81-dependent pathway, which is, by inference from the yeast data, interference-independent [8]. In the absence of MUS81 in *Mus81^−/−^* spermatocytes, MLH1–MLH3 would not be repressed at these CO sites, thus increasing overall MLH1 focus number, and decreasing the amount of interference between MLH1 foci, while maintaining the eventual number of chiasmata (see below). MUS81-MLH1-MLH3 crosstalk may be mediated by BLM, as BLM is known to interact with MUS81 in somatic cells [51] and with key components of the DNA mismatch repair family, including MLH1 [52]. These data predict that interference is laid down prior to MLH1–MLH3 accumulation, and remains in place regardless of the pathway by which COs are processed. This represents the first data on interference decision and timing in mouse spermatocytes and is supported by data concerning the role of MUS81-EME1 in cleaving HJs *in vitro* and *in vivo*. MUS81 readily cleaves non-HJ substrates *in vitro* [12],[45],[53],[54],[55], and can efficiently catalyse symmetrical and coordinated cleavage of intact HJs *in vitro* [14],[16]. The potential importance of this intact HJ resolution activity is supported by indirect evidence that MUS81-EME1 cleaves *HJ in vivo*, at least in fission yeast [12],[15], but has yet to be demonstrated definitively in other eukaryotes.

A third possibility is that the extra MLH1 foci seen in *Mus81^−/−^* mice are not representative of extra COs, but instead of aberrant repair structures that require MUS81 protein to be repaired correctly, or which arise as a result of failure of MUS81-driven processes (as discussed above). From our data, it is difficult to say whether this is the case. The persistent BLM and RAD51 foci seen in the mutant spermatocytes indicate repair defects, rather than crossover anomalies yet, at the same time, since we see increased MLH3 foci in addition to the increase in MLH1 foci, it is equally likely that these structures represent nascent COs rather than unrepaired breaks.

In *Mus81^−/−^* mice, the number of chiasmata was not different from that of WT mice, despite the increase in MLH1 foci at pachynema. However chiasma numbers were significantly reduced with respect to MLH1 focus number in *Mus81^−/−^* pachytene cells, indicating either that some MLH1 foci are lost before they are resolved as chiasmata or that cells with higher number of MLH1 foci undergo apoptosis following pachynema. Cells with MLH1–MLH3 foci too close together may be subject to additional processing by downstream factors limiting the crossover number, such as EXO1 or BLM (Sgs1 in yeast) [56],[57] which, we suggest, would normally overlook interference-independent MUS81 foci. Unfortunately, analysis of the localization of EXO1 is precluded by the absence of an antibody that is functional on chromosome spreads. However BLM appears to persist into late pachynema in *Mus81* spermatocytes and may account for this additional surveillance mechanism. Oh *et al.* [57] have proposed that Sgs1 in yeast functions to suppress closely spaced COs by preventing the formation of complex recombination intermediates involving multiple chromatids. In this model, Mlh1–Mlh3 complexes act to promote inter-homolog strand invasion, in part by antagonizing Sgs1. At the same time, Sgs1 acts to disassemble complex recombination intermediates that might include those resulting from closely spaced DSB processing events. Given these suggestions, the current data indicate that the absence of MUS81 in mice results in closely spaced interference-independent MLH1–MLH3 events that then become the target for directed BLM action. We suggest, therefore, that CO control in the mouse involves complex integration between MUS81 and MLH1–MLH3, perhaps mediated by BLM.

The current study provides strong evidence that COs in mammals are controlled by at least two pathways, and that MUS81 is responsible for processing a distinct subset of these events. Whether this control is mediated early in prophase I, at the level of RAD51 acquisition, or later, upon accumulation of MLH1/MH3, remains to be seen. Clearly, however, the function of MUS81 in meiosis differs between eukaryotic species and also possibly betweeen its function in the context of replication and meiosis. Continued cross-species analysis of MUS81 action will help to elucidate the nature of its activity in these diverses processes.

## Materials and Methods

### Mouse Breeding

*Mus81* knockout mice were generated as previously described [25]. Hybrid mice were bred with C57Bl/6 for several generations, until mice were over 85% C57Bl/6 background. Analyses were performed both on *Mus81^−/−^* mutants and aged-matched wild-type control mice. Analyses were repeated on WT, heterozygote and mutant littermates. *Mlh3* and *Msh4* knock-out mice have been previously described [22],[58].

### Sperm Counts

Epidiymides were removed from either mutant or WT adult mice, placed in human tubule fluid (HTF) culture medium containing BSA (Specialty media), ripped open using micro forceps and the contents squeezed into the medium. The spermatozoa were cultured for 20 minutes at 32°C, then a 20 µl aliquot was removed and fixed in 480 µl 10% formalin. The fixed cells were gently mixed then intact spermatozoa counted using a hemocytometer.

### Histology

Adult mice were subjected to either perfusion fixation with Bouins fixative or the testes were removed and fixed in 10% buffered formalin for 12 hours at 4°C. Paraffin-embedded tissue was sectioned at 4 µm and processed for Haematoxylin and Eosin staining or immunohistochemical analyses using standard methods.

### Chromosome Spread Analysis

Testes were removed from mutant and WT mice aged day 12 pp, day 14 pp, day 17 pp and day 20 pp for the meiotic time course analysis, as well as adult mice for the focus counts, and processed as previously described [59]. Briefly, testes were removed and decapsulated into hypotonic sucrose extraction buffer (HEB, containing 1.7% sucrose) and left on ice for 30 min–1 hr. Tubules were chopped on glass depression slides in a bubble of 0.03% sucrose and added to slides coated in 1% paraformaldehyde. For analysis of female chromosome spreads, ovaries were removed from day e18.5 to day 0.5 pp females, briefly soaked in HEB, minced in 0.03% sucrose and added to a bubble of paraformaldehyde on a well slide. Slides were slow dried and subjected to immunofluorescent analyses.

### Immunofluorescence and Immunohistochemistry

Slides were processed as described previously [60] using antibodies generated in this lab [59], generously donated by colleagues and available commercially. Primary antibodies used included polyclonal rabbit-anti-RAD51 (1∶500, Oncogene research products), mouse-anti-MLH1 (1∶50, Santa Cruz), and rabbit anti-MSH4 (1∶10) [58].

Immunohistochemistry was performed on formalin-fixed sections using rat hybridoma supernatant against germ cell nuclear antigen-1 (GCNA-1) [61] for staining of early spermatocytes or TUNEL staining (Chemicon) to detect cells undergoing apoptosis.

### Statistical Analyses

Interfocus distances were collected from all 19 chromosomes from late pachytene cells using the computer application MicroMeasure version 3.01 (available for free download via the Internet at http://www.colostate.edu/Depts/Biology/MicroMeasure). The interference parameter, *v*, was estimated using two independent methods. In the first, *v* was estimated by fitting the observed frequency distribution of inter-focus distances, for measurements only from chromosome 1 and 2, to the gamma distribution by the maximum likelihood method using the GENSTAT software package (VSN International, Hemel Hempstead, UK) as described previously [37]. The second method utilized data from all chromosomes, with distances measured directionally, beginning at the centromere. Distances from the last focus to the end of the chromosome were considered right-censored observations, and the inter-focus distances non-censored observations. Thus, every chromosome from which data was collected had one censored inter-focus distance measurement. Since most chromosomes contain either one or two foci, most of the inter-focus distances are censored. The shape and scale parameters were estimated by maximum likelihood, with the censoring being handled using the EM algorithm for exponential families [62] and fitted to a gamma distribution.

Testis weights, spermatozoa numbers, TUNEL analysis, immunofluorescent focus counts (RAD51, MSH4, MLH1) and diakinesis spread counts were all analyzed for statistical significance by using an unpaired t-test.
